# Epigenetic targeting of neuropilin-1 prevents bypass signaling in drug-resistant breast cancer

**DOI:** 10.1038/s41388-020-01530-6

**Published:** 2020-10-30

**Authors:** Ammara Abdullah, Saeed Salehin Akhand, Juan Sebastian Paez Paez, Wells Brown, Li Pan, Sarah Libring, Michael Badamy, Emily Dykuizen, Luis Solorio, W. Andy Tao, Michael K. Wendt

**Affiliations:** 1 Department of Medicinal Chemistry and Molecular Pharmacology, Purdue University, West Lafayette, IN 47907 USA; 2 Department of Biomedical Engineering, Purdue University, West Lafayette, IN 47907 USA; 3 Purdue University Center for Cancer Research, Purdue University, West Lafayette, IN 47907 USA

**Keywords:** Breast cancer, Cancer imaging, Targeted therapies, Oncogenes, Target identification

## Abstract

Human epidermal growth factor receptor 2 (*HER2*)-amplified breast cancers are treated using targeted antibodies and kinase inhibitors, but resistance to these therapies leads to systemic tumor recurrence of metastatic disease. Herein, we conducted gene expression analyses of HER2 kinase inhibitor-resistant cell lines as compared to their drug-sensitive counterparts. These data demonstrate the induction of epithelial–mesenchymal transition (EMT), which included enhanced expression of fibroblast growth factor receptor 1 (FGFR1) and axonal guidance molecules known as neuropilins (NRPs). Immunoprecipitation of FGFR1 coupled with mass spectroscopy indicated that FGFR1 forms a physical complex with NRPs, which is enhanced upon induction of EMT. Confocal imaging revealed that FGFR1 and NRP1 predominantly interact throughout the cytoplasm. Along these lines, short hairpin RNA-mediated depletion of NRP1, but not the use of NRP1-blocking antibodies, inhibited FGFR signaling and reduced tumor cell growth in vitro and in vivo. Our results further indicate that NRP1 upregulation during EMT is mediated via binding of the chromatin reader protein, bromodomain containing 4 (BRD4) in the NRP1 proximal promoter region. Pharmacological inhibition of BRD4 decreased NRP1 expression and ablated FGF-mediated tumor cell growth. Overall, our studies indicate that NRPs facilitate aberrant growth factor signaling during EMT-associated drug resistance and metastasis. Pharmacological combination of epigenetic modulators with FGFR-targeted kinase inhibitors may provide improved outcomes for breast cancer patients with drug-resistant metastatic disease.

## Introduction

The ErbB family members ErbB1 (epidermal growth factor receptor (EGFR)) and ErbB2 (human epidermal growth factor receptor 2 (HER2)) are intensively studied receptor tyrosine kinases (RTKs) with fundamental roles in cancer. In breast cancer, *HER2* becomes amplified and patients with HER2^+^ tumors can respond to treatment with the monoclonal antibodies pertuzumab and trastuzumab. However, acquired and inherent resistance remains a major clinical problem in this breast cancer subtype, particularly in the metastatic setting. In addition, expression of cytokeratin 5/6 and EGFR are major factors in defining the basal-like subtype of breast cancer and further decrease the already abysmal prognosis of this aggressive form of disease [1, 2]. Unlike HER2, monotherapies directed toward EGFR or in conjunction with other adjuvant therapies do not provide clinical benefit to breast cancer patients, suggesting a more active mode of resistance [3–5]. The development of EGFR/HER2 dual targeting kinase inhibitors such as lapatinib and the covalent “pan ErbB” inhibitors neratinib and afatinib do offer more tools for clinical inhibition of ErbB signaling, but recent studies suggest that even these compounds are subject to the drawbacks of therapeutic resistance via bypass signaling [6, 7].

Analysis of The Cancer Genome Atlas dataset indicates that 12.5% of 1105 invasive breast tumors contain amplification of the *FGFR1* locus [8]. Furthermore, elevated expression of FGFR1 is associated with poor prognosis of breast cancer patients [9, 10]. Recent studies further indicate that *FGFR1* can become amplified de novo in metastases as compared to the primary tumors from which they were derived [11]. We and others have previously demonstrated that expression of FGFR1 is also enhanced through the processes of epithelial–mesenchymal transition (EMT). In addition to increased expression of the receptor, additional EMT-associated events such as changes in cadherin expression and upregulation of integrins are critical in facilitating ligand-mediated FGFR signaling [12, 13]. Upregulation of FGFR in the context of EMT is established as a robust resistance mechanism for tumor cells that were originally sensitive to EGFR- and HER2-targeted agents [6, 14]. Several different kinase inhibitors against FGFR have been developed, and we and others have demonstrated the in vivo efficacy of these molecules in delaying the growth of drug-resistant and metastatic breast cancer [15–17].

Neuropilin-1 (NRP1) and NRP2 are non-enzymatic molecules that act in conjunction with the plexin family of receptors by binding the class 3 family of semaphorin ligands to facilitate axonal migration of developing neurons [18, 19]. In addition, NRP1/2 bind vascular endothelial growth factor (VEGF) and participate in the growth and migration of endothelial cells [20]. In addition to binding semaphorins and VEGF, NRP1 has been suggested to act as a co-receptor for several other RTKs [21, 22]. In fact, recent studies point to a role for NRP1 in facilitating bypass pathway signaling during drug resistance [23, 24]. These findings supported the construction of a therapeutic antibody designed to inhibit the ability of NRP1 to bind VEGF. Unfortunately, clinical evaluation of NRP1-targeted antibodies has thus far failed to produce benefits for patients with advanced tumors [25, 26].

Herein, we demonstrate that FGFR1 and NRP1 are upregulated in several independent models of EMT-associated acquisition of resistance to ErbB-targeted kinase inhibitors. These molecules physically interact to produce an active signaling complex. Overall, the current study sought to address the hypothesis that EMT-mediated upregulation of NRP1 expression facilitates resistance to ErbB-targeted therapies via bypass RTK signaling.

## Results

### Identification of EMT-associated mediators of ErbB inhibitor (ErbBi) resistance

**Fig. 1:**
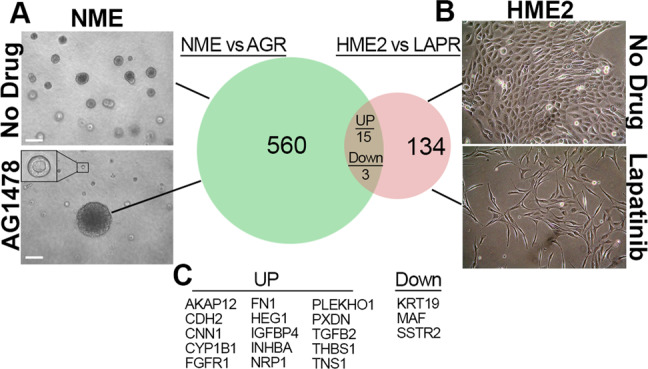
Identification of shared mediators of ErbBi resistance. **a** Aberrant growth of normal murine mammary gland cells transformed by overexpression of EGFR (NME cells) was normalized by the addition of the EGFR inhibitor AG1478 (1 µM). (Inset shows a typical small, acinar structure.) After 14 days of drug treatment, an AG1478-resistant (AGR) colony from these 3D cultures was isolated and characterized by differential gene expression analyses in comparison to the parental NME cells. **b** Human mammary epithelial cells transformed by overexpression of HER2 (HME2 cells) were cultured in the presence of lapatinib (1 µM) until lapatinib-resistant (LAPR) cells emerged. The LAPR cells were characterized by differential gene expression as compared to parental HME2 cells. **c** The differentially expressed genes in both ErbBi resistance models were compared. Genes similarly regulated by >2-fold upon acquisition of resistance to either AG1478 or lapatinib are listed.

**Fig. 2:**
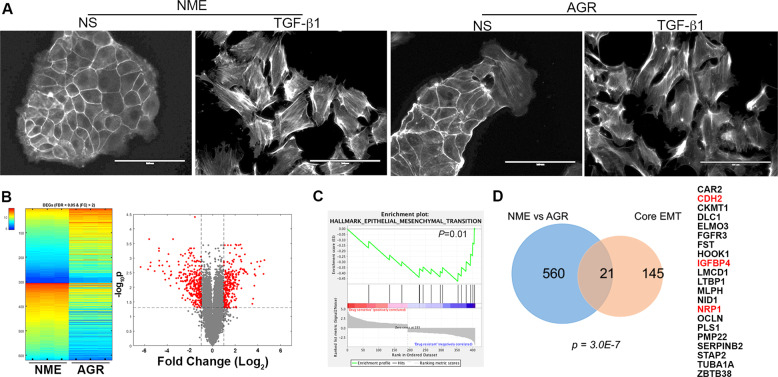
Drug-resistant cells isolated from 3D culture have undergone EMT. **a** Phalloidin staining to visualize the actin cytoskeleton in the NME and AGR cell lines under non-stimulated (NS) conditions and after 72 h of TGF-β1 (5 ng/ml) stimulation. **b** Heatmap and volcano plot visualizing differentially expressed genes (DEGs) between the drug-sensitive (NME) and AG1478-resistant (AGR) cell lines. The triplicate microarray analyses resulting in upregulated (top) and downregulated (bottom) genes with a fold change (FC) of >2 and a false discovery rate (FDR) of <0.05 are shown on the heatmap. These DEGs are also shown in red on the volcano plot. **c** Gene set enrichment analysis comparing the DEGs identified in the AGR cells to the hallmark-EMT gene set. This comparison resulted in the indicated *P* value. **d** Venn diagram indicating the number of genes similarly regulated in AGR vs. NME DEG set as compared to the core-EMT gene signature. The significance of overlap was calculated using a hypergeometric model resulting in the indicated *P* value. The 21 shared genes are listed on the right. The genes also regulated in the LAPR gene set described in Fig. 1 are indicated in red.

In order to identify mediators of resistance to EGFR- and HER2-targeted kinase inhibitors, our lab has developed numerous models of drug resistance using mammary epithelial cells of both human and murine origin. For instance, normal murine mammary gland cells (NMuMG) can be transformed by overexpression of EGFR, we have termed these NME cells [27]. The transformed phenotype of NME cells manifests in 3D culture as large filled structures, as opposed to the hollow acinar structures that are characteristic of NMuMG cells [28]. Formation of acinar structures by NME cells can be restored by addition of the EGFR inhibitor AG1478 (Fig. 1a). Using this approach, we observed and were able to isolate an AG1478-resistant (AGR) 3D colony (Fig. 1a). Following expansion of these cells, their maintained resistance to EGFR inhibition was verified, as was the ability of the EGFR inhibitor, AG1478, to block EGF-mediated cell signaling events (Supplementary Fig. S1). These data suggested activation of bypass pathways as a resistance mechanism, as opposed to secondary resistance mutations in EGFR. Therefore, we utilized global gene expression analyses to characterize the AGR cells in comparison to their AG1478-sensitive, NME counterparts (Fig. 1a; GSE140978). Differentially expressed genes (DEGs) found in this model were compared to our recently characterized model of acquired resistance to lapatinib [29]. Here, human mammary epithelial (HMLE) cells transformed by overexpression of HER2 (HME2) were treated with lapatinib for 4 weeks in 2D culture at which point extremely mesenchymal, lapatinib-resistant (LAPR) cell populations emerged (Fig. 1b). After culture for several passages in the absence of drug, the parental HME2 cells and two independently isolated LAPR populations were transiently (96 h) exposed to lapatinib and short- and long-term changes in gene expression were characterized using the NanoString PanCancer Progression panel (Supplementary Table 1). Comparison of the AGR and LAPR datasets yielded an ErbBi resistance signature of genes whose expression were similarly regulated by >2-fold in both AGR and LAPR cells (Fig. 1c). Since the AGR cell line was isolated from 3D culture conditions, we first sought to determine if the phenotype that manifests under these conditions was unique from 2D culture-derived resistance models. Culture of the AGR cells in 2D conditions resulted in a cell morphology that maintained cell–cell junctions, but was distinct from the parental NME cells (Fig. 2a). The mesenchymal morphology of the AGR cells could be further enhanced by treatment with transforming growth factor-β1 (TGF-β1) (Fig. 2a). Consistent with these morphological changes, the DEGs in the AGR line did result in a significant gene set enrichment analysis when compared to the hallmark-EMT gene signature (Fig. 2b, c). Finally, we compared the 581 DEGs from the AGR cell line to the previously established 146 core-EMT gene signature. The core-EMT genes are similarly modulated by several different master regulators of EMT such as snail, twist, goosecoid, stimulation with TGF-β1, and directed depletion of E-cadherin (E-Cad) [30]. Using this approach, we also found a highly significant correlation between the core-EMT signature and the AGR DEG list (Fig. 2d). Therefore, we conclude that similar to 2D-derived models, isolation of drug-resistant cell lines from 3D culture also results in stable induction of EMT.

Cross-referencing the 21 EMT-associated genes in the AGR DEG list with our ErbBi resistance signature established in Fig. 1 demonstrated three similarly upregulated genes, *CDH2*, *IGFBP4* (insulin-like growth factor binding protein 4), and *NRP1* (Fig. 2d). Enhanced expression of *IGFBP4* is consistent with inhibition of insulin growth factor receptor signaling, and therefore was not further pursued in this study as a targetable ErbBi resistance mechanism. In contrast, CDH2, which encodes for neuronal-cadherin, and NRP1 have both been described as co-receptors that can facilitate growth factor signaling and drug resistance [13, 31]. Analysis of additional resistance models using a cell line derived from HME2 bone metastases (HME2-BM) similarly demonstrated upregulation of NRP1 upon acquired resistance to the second-generation covalent ErbB-targeting drugs, afatinib and neratinib (Supplementary Fig. S2a–c). Moreover, analysis of ErbBi-treated HER2^+^ patient samples demonstrated that higher levels of NRP1 expression is associated with decreased survival (Supplementary Fig. S2d) [32]. Therefore, the mechanistic role of NRP1 in facilitating resistance to ErbB-targeted agents was pursued for further study.

### Neuropilins interact with the FGFR1 signaling complex

**Fig. 3:**
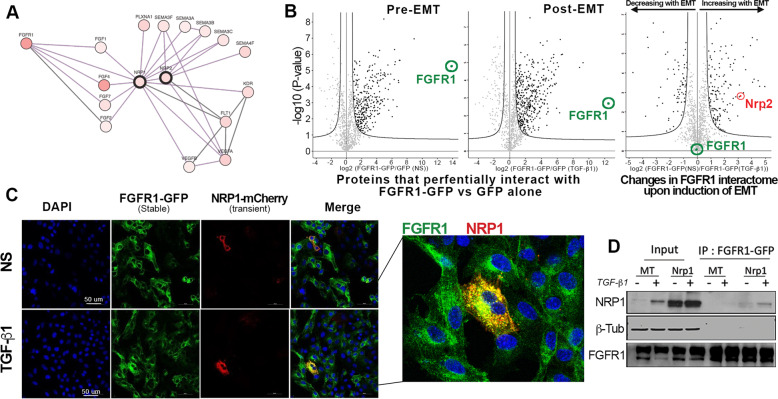
NRP1 physically interacts with FGFR1 following induction of EMT. **a** String plot created using human protein reference database showing NRP1-interacting proteins. **b** Volcano plots visualizing mass spectrometry analysis of proteins that preferentially co-immunopreciptiated with FGFR1-GFP as compare to GFP alone in NMuMG cells under non-stimulated (pre-EMT) conditions or following a 72-h stimulation with TGF-β1 (5 ng/ml) (post-EMT). Comparison of these two datasets yielded changes in the FGFR1 interactome upon EMT induction. Significant protein interactions are shown as black dots. **c** NMuMG cells stably expressing FGFR1-GFP cells were transiently transfected with NRP1-mcherry and similarly treated with TGF-β1 (5 ng/ml) for 72 h. Fluorescent images were taken using a confocal microscope. **d** NRP1 or Empty (MT) vectors were expressed in NMuMG-FGFR1-GFP cells and cells were treated with TGF-β1 (5 ng/ml) for 72 h. FGFR1-GFP was immunoprecipitated using the GFP-trap assay and precipitates were directly probed for NRP1. Data in **c**, **d** are representative of at least three independent assays.

Analysis of the human protein reference database for proteins that interact with NRP1 revealed the known interactions with NRP2, several plexin and semaphorin molecules, and VEGF receptors (Fig. 3a). However, in vitro biochemical experiments using an optical biosensor-based binding assay also suggested that NRP1 can physically interact with FGFs and FGFR1 (Fig. 3a) [21]. Interestingly, FGFR1 was part of our ErbBi resistance signature from Fig. 1 and our previous studies functionally associate FGFR1 with EMT-driven resistance to the EGFR/HER2 inhibitor, lapatinib [29]. To characterize the FGFR1 interactome, we conducted immunoprecipitation (IP) of a GFP-tagged FGFR1 before and after induction of EMT, and associated proteins were identified by mass spectroscopy (Fig. 3b). These analyses confirmed many known FGFR1 interaction partners including E-Cad and neural cell adhesion molecule (Supplementary Tables 2–4). In addition, an NRP2 peptide was identified as a molecule whose interaction with FGFR1 was specifically enhanced upon induction of EMT (Fig. 3b). Next, we transiently expressed an mCherry-tagged NRP1 construct in cells stably expressing FGFR1-GFP. Confocal microscopy revealed that FGFR1 robustly interacted with NRP1 in cytoplasmic puncta, particularly following induction of EMT (Fig. 3c). These data were confirmed by direct co-IP assays showing that FGFR1 and NRP1 form a physical complex that is enhanced upon induction of EMT (Fig. 3d).

### NRP1 and FGFR1 are required for pulmonary tumor growth

**Fig. 4:**
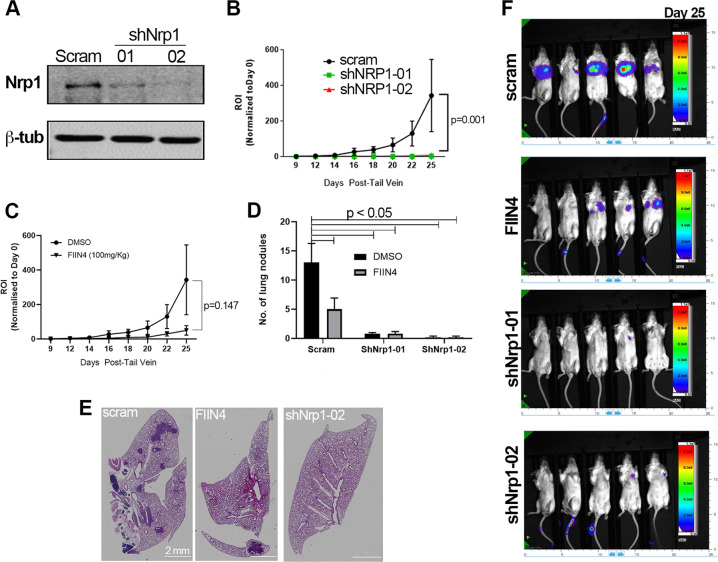
Depletion of NRP1 prevents pulmonary tumor growth. **a** Immunoblot analyses of control (scram) and NRP1-depleted (shNRP1-01, shNRP1-02) D2.A1 cells. **b** Bioluminescent quantification of pulmonary tumor growth in animals injected via the lateral tail vein with control D2.A1 cells (scram) and those depleted for NRP1. Data are the mean ± SE of five mice per group at the indicated time points resulting in the indicated *P* value. **c** Bioluminescent quantification of pulmonary tumor growth following lateral tail vein injection with the D2.A1 cells. Animals were either treated with vehicle control (DMSO) or a covalent inhibitor of FGFR (FIIN4) at 100 mg/kg/Q.O.D. Data are the mean ± SE of five mice per group at the indicated time points resulting in the indicated *P* value. **d** Animals were injected and treated as described in **b**, **c** and upon necropsy, the average number of pulmonary tumor nodules per mouse were quantified for control, FIIN4-treated, and NRP1-depleted groups. **e** Representative H&E sections of single pulmonary lobes of control, FIIN4-treated and NRP1-depleted tumors. **f** Representative bioluminescent images for the indicated groups at the time of necropsy.

In addition to initial response and acquisition of resistance, inherent resistance to ErbBi therapies is a major clinical issue when attempting to treat metastatic breast cancer [33]. Our lab has previously established that the D2-HAN series of metastatic progression serves as a model of inherent resistance to ErbBi [15]. As opposed to the non-metastatic D2.OR cells, the D2.A1 cells, their isogeneic and metastatic counterparts, express lower levels of HER2 and EGFR. Neratinib dose–response assays confirmed that the D2.A1 cells, as compared the D2.OR cells, are indeed inherently resistant to this second-generation, covalent ErbBi (Supplementary Fig. S3). To evaluate the role of NRP1 in facilitating non-ErbB signaling, we depleted NRP1 expression from the D2.A1 cells using two independent short hairpin RNA (shRNA) targeting sequences (Fig. 4a). Depletion of NRP1 decreased in vitro cell proliferation and dramatically prevented D2.A1 tumor formation within the pulmonary microenvironment, following tail vein inoculation of equal numbers of viable cells (Fig. 4b and Supplementary Fig. S4a). Specific targeting of FGFR using FIIN4, a covalent inhibitor of FGFR kinase activity, failed to significantly reduce pulmonary tumor growth as determined by bioluminescent imaging. However, a significant reduction in pulmonary tumor nodules was observed in FIIN4-treated mice (Fig. 4c–f). Depletion of NRP1 inhibited pulmonary tumor formation so profoundly that evaluation of FIIN4 treatment in combination with NRP1 depletion was not possible (Fig. 4d). In addition, we utilized YW107.4, an antibody developed to therapeutically neutralize NRP1 [34]. In vitro use of this antibody was able to significantly inhibit FGF2-induced cell growth (Supplementary Fig. S4b). However, in stark contrast to the results obtained upon genetic depletion of NRP1, in vivo use of YW107.4 enhanced the growth of D2.A1 pulmonary tumors as compared to control animals (Supplementary Fig. S4c, d). Taken together, these data suggest that NRP1 is a critical mediator of ErbB-independent pulmonary tumor progression, but antibody therapies may not be an effective means of targeting this molecule.

### NRP1 regulates RTK signaling and growth of breast cancer cells

**Fig. 5:**
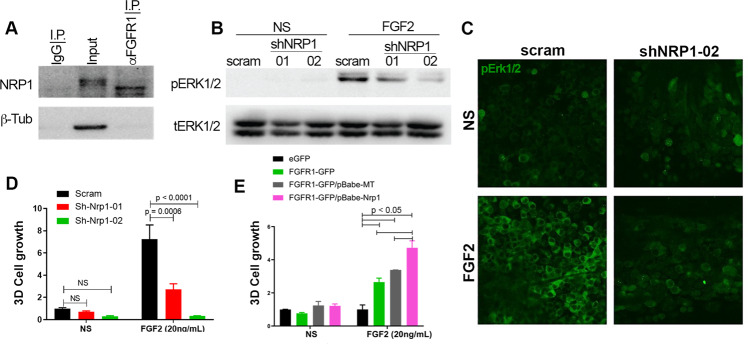
NRP1 facilitates FGF2-mediated signaling and cell growth. **a** Endogenous FGFR1 was immunoprecipitated from D2.A1 whole-cell lysates. These precipitates were probed by immunoblot with antibodies for NRP1. Total levels of NRP1 (input) and use of a non-specific antibody (IgG) are shown as controls. **b** Immunoblot analyses showing differential phosphorylation of ERK1/2 (pERK1/2) upon FGF2 (20 ng/ml) stimulation in control (scram) and NRP1-depleted (shNRP1-01 and -02) D2.A1 cells. Expression of total ERK1/2 (tERK1/2) served as a loading control. **c** Immunofluorescent staining for phosphorylated-ERK1/2 (pERK1/2) in control (scram) and NRP1-depleted (shNRP1-02) D2.A1 cells grown on 3D culture scaffolds. Cells were not stimulated (NS) or stimulated with FGF2 (20 ng/ml) for 10 min. Data in **a**–**c** are representative of at least three independent analyses. **d** Bioluminescent quantification of control (scram) and NRP1-depleted (shNRP1) D2.A1 cells growing under 3D culture conditions in the presence or absence (NS) of FGF2 (20 ng/ml). **e** Bioluminescent quantification of NMuMG cells stably expressing control vectors (eGFP and empty (MT)) or FGFR1-GFP and NRP1 in 3D culture in the presence or absence (NS) of FGF2 (20 ng/ml). For **d**, **e**, readings were taken 6 days after plating and are the mean ± SE of two independent experiments completed in triplicate resulting in the indicated *P* values.

Consistent with our overexpression systems, further characterization of FGFR1 and NRP1 in the D2.A1 model confirmed a physical interaction between these endogenously expressed proteins (Fig. 5a). Depletion of NRP1 decreased the ability of FGF2 to induce ERK1/2 phosphorylation as determined by immunoblot under 2D culture conditions (Fig. 5b). We have previously established that FGFR1 interacts with integrins and the presence of an extracellular matrix enhances the response to FGF2 as compared to 2D culture conditions [12]. Therefore, we transiently stimulated D2.A1 cells with FGF2 as they grew on our tessellated cell-culture system that allows for cells to grow on suspended fibrillar fibronectin independent of polymeric or gel-based support [35]. Immunofluorescent staining clearly indicated that lack of NRP1 prevented the ability of FGF2 to induce ERK1/2 phosphorylation even when cells were growing in the context of an extracellular matrix (Fig. 5c). As a quantitative output of these signaling data, we were able to demonstrate that depletion of NRP1 prevented FGF2-induced cell growth within 3D cultures (Fig. 5d). To complement this depletion approach, we stimulated FGFR1-overexpressing NMuMG cells with FGF2, in the presence or absence of NRP1 overexpression. This approach demonstrated that optimal growth response to FGF2 in 3D culture was only achieved when both NRP1 and FGFR1 were present (Fig. 5e). Expanding these observations beyond FGF2, we also observed that depletion of NRP1 similarly prevented D2.A1 cell growth induced by hepatocyte growth factor (HGF) and platelet-derived growth factor (PDGF) (Supplementary Fig. S5).

### EMT-induced NRP1 expression can be blocked by inhibition of bromodomain containing 4 (BRD4)

**Fig. 6:**
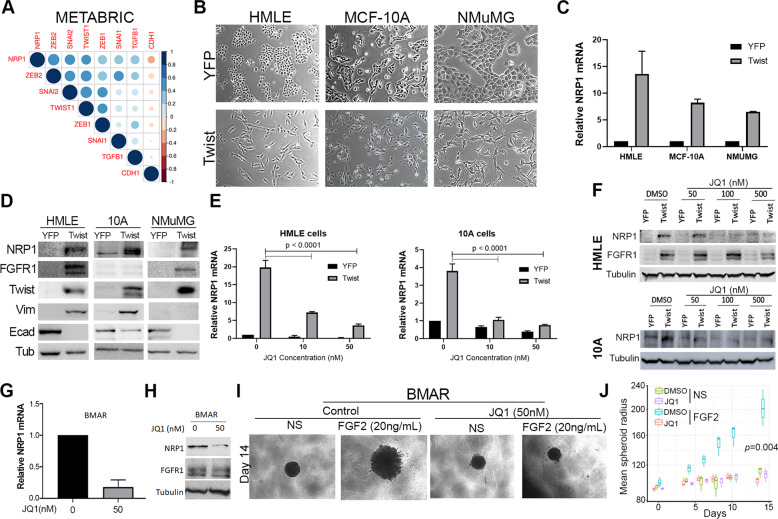
Inhibition of BRD4 decreases NRP1 expression. **a** Correlation between EMT transcription factors and NRP1 gene expression in the METABRIC dataset. **b** Phase-contrast microscopy showing the epithelial and mesenchymal phenotypes of control (YFP) and Twist-overexpressing HMLE, MCF-10A, and NMuMG cells. **c** RT-PCR showing NRP1 upregulation following Twist overexpression. Data are normalized to NRP1 levels in the control (YFP) cells for each cell type and are the mean ± SE of duplicate experiments completed in triplicate. **d** Immunoblot analyses showing expression of NRP1 and other EMT proteins in response to Twist overexpression. **e** RT-PCR showing NRP1 in control (YFP) and Twist-overexpressing cells following 7 days of treatment with the indicated concentrations of JQ1. Data are the mean ± SE of triplicate experiments resulting in the indicated *P* values. **f** Immunoblot analyses of NRP1 expression in control (YFP) and Twist-overexpressing HMLE and MCF-10A cells following 7 days of treatment with the indicated concentrations of JQ1. **g** RT-PCR analyses of NRP1 expression in afatinib-resistant (BMAR) cells following 7 days of treatment with JQ1 (50 nM). Data are the mean ± SE of triplicate experiments. **h** Immunoblot analyses of FGFR1 and NRP1 expression in BMAR cells following 7 days of treatment with JQ1 (50 nM). Expression of tubulin served as a loading control. **i** Phase-contrast images showing 3D spheroid growth of BMAR cells in response to FGF2 (20 ng/ml) in the presence or absence of JQ1 (50 nM). **j** Measurement of BMAR spheroid radius at the indicated time points, under the conditions described in **i**. Data are represented as a boxplot including the measurement of at least three images per condition and are representative of two independent experiments.

Our observation that antibody-mediated targeting of cell surface NRP1 fails to significantly inhibit pulmonary tumor growth is consistent with the lack of clinical efficacy observed using this approach (Supplementary Fig. S4) [36]. Given these data and our observation that NRP1 interacts with FGFR1 throughout the cytoplasm, we sought to identify and pharmacologically target transcriptional mediators of EMT-induced NRP1 overexpression. To do this, we analyzed the molecular taxonomy of breast cancer international consortium (METABRIC) dataset for transcriptional regulators that correlated with NRP1 expression. Consistent with NRP1 being part of the core-EMT signature, we observed the EMT-associated transcription factors SNAIL1 and 2, Zeb1 and 2, and Twist1 to strongly correlate with NRP1 expression (Fig. 6a). To directly test the ability of these factors to drive NRP1 expression, we overexpressed Twist1 in three mammary epithelial cell lines (Fig. 6b). This approach clearly indicated that Twist1 was sufficient to drive a morphological change in these cells and upregulate NRP1 expression at both the mRNA and protein levels (Fig. 6b–d). To better understand the mechanisms of Twist-mediated NRP1 expression, we explored its defined interaction with the chromatin reader protein BRD4. To do this, we analyzed chromatin IP-sequencing data for H3K27 acetylation, a chromatin modification associated with BRD4 binding and transcriptional activation (Supplementary Fig. S6) [37]. These data indicated that H3K27 was acetylated and bound by BRD4 immediately upstream of *NRP1* transcriptional start (Supplementary. Fig S6a). Furthermore, RNA-sequencing data (GSE63584 and GSE53222) suggest that treatment with JQ1, a BRD4 inhibitor, is capable of inhibiting expression of NRP1 in both control and Twist-overexpressing cells (Supplementary Fig. S6b, c) [38]. Consistent with the notion that BRD4 is required for the induction of NRP1 during EMT and drug resistance, we found that JQ1 inhibited NRP1, but not FGFR1, expression in Twist-expressing cells, ErbBi-resistant BMAR, LAPR, and D2.A1 cells (Fig. 6e–h; Supplementary Fig. S7a–d). Not only that, but pretreatment of JQ1 inhibited the ability of FGF2 and PDGF to induce phosphorylation of ERK1/2 (Fig. S7e). Finally, using our recently described 3D tumor spheroid model, we were able to demonstrate that subtoxic doses of JQ1 effectively blocked the ability of FGF2 to induce the growth and invasion of ErbBi-resistant and metastatic cells (Fig. 6i–j and Supplementary Fig. S8a–d) [39].

## Discussion

**Fig. 7:**
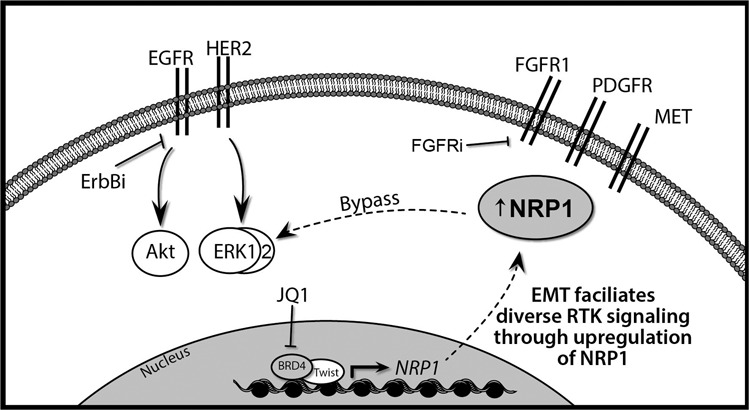
EMT facilitates diverse RTK signaling through the upregulation of FGFR1 and NRP1. Transformation and breast cancer progression are driven by ErbB signaling through EGFR and HER2. These signaling pathways can be effectively blocked through the use of several small molecules that directly act on these RTKs, collectively referred to here as ErbB inhibition (ErbBi). Induction of epithelial–mesenchymal transition (EMT) is a shared mechanism of acquired resistance to ErbBi. Through this process tumor cells originally driven by ErbB signaling upregulate FGFR1 and NRP1. FGFR1 can be directly targeted through the use of small molecule kinase inhibitors (FGFRi). In addition to FGFR1, NRP1 can also enhance signaling from other RTKs that are present such as PDGFR and the MET receptor. Twist-mediated expression of NRP1 during EMT requires BRD4 binding to acetylated H3K27. Therefore, the use of BET inhibitors such as JQ1 in conjunction with targeted inhibition of FGFR may present an effective means to block ErbBi bypass signaling.

Acquisition of resistance to ErbB-targeted therapies in metastatic breast cancer patients constitutes a major clinical challenge for the HER2^+^ and basal subtypes. Identification of targetable underlying mechanisms of resistance is crucial to improve therapeutic strategies for these patients. Our gene expression analyses of mouse and human models of acquired ErbBi resistance demonstrated enhanced expression of NRP1 and FGFR1. FGFR1 is an established modulator of tumor progression and resistance to currently used therapeutics [29, 40, 41]. NRP1 is a multifunctional protein involved in axon guidance, VEGF signaling, ECM interaction, and TGF-β signaling [42–45]. Therefore, we sought to further investigate the role of NRP1 in facilitating bypass RTK signaling during ErbBi resistance and metastasis (Fig. 7).

Using an additional discovery approach, our co-IP mass spectrometry analyses indicated that FGFR1 interacts with NRPs and that this interaction is enhanced once cells undergo EMT. An intuitive hypothesis was that similar to its functional interaction with PLEXIN and VEGF receptors, the interaction of NRP1 with FGFR1 would similarly take place on the cell surface to facilitate ligand response. However, our confocal imaging of cells expressing FGFR1 and NRP1 suggest that the interaction between FGFR1 and NRP1 takes place to a large extent, while both molecules are in the cytoplasm. Indeed, we recently demonstrated that when cells undergo EMT, FGFR1 exits cell–cell junctions in favor of a cytoplasmic localization [12]. Furthermore, mitochondrial functions of both NRP1 and FGFR1 have been previously identified [46, 47]. While beyond the scope of the current study, identifying the functional significance of an NRP:FGFR1 complex in the mitochondria is an active area of research in our group. Together with the known drawbacks of targeting VEGF signaling in metastatic breast cancer, our findings may, in part, serve to explain the clinical inability of NRP1 antibody blockade to effectively inhibit metastatic tumor progression [36, 48]. Importantly, our syngeneic in vivo data using a murine NRP1-blocking antibody similarly produced confounding results as compared to the dramatic inhibition of pulmonary tumor growth observed upon shRNA-mediated depletion of total levels of NRP1. While the reasons for this result remain to be definitively determined, previous studies have demonstrated that anti-NRP1 antibodies first saturate host pulmonary tissues prior to binding to tumor cells [49]. Anti-NRP1 antibody binding to pulmonary vasculature could alter vasculature structure, an event known to promote the outgrowth of disseminated tumor cells [50, 51].

Given the failure of antibody-mediated therapies targeting NRP1, we sought to explore the mechanisms by which NRP1 expression becomes enhanced during EMT, drug resistance, and metastasis. Since NRP1 does not have an enzymatic function, we reasoned that pharmacological normalization of its expression could yield therapeutic benefit. Exploration of existing datasets from both patient samples and various EMT-induction models clearly indicated that the master EMT transcription factor Twist1 is capable of driving NRP1 expression [37, 38]. Importantly, our studies also implicate the requirement of chromatin remodeling and BRD4 in mediating NRP1 overexpression during EMT and drug resistance (Fig. 7). The use of JQ1 at concentrations well below induction of non-specific cytotoxicity was sufficient to normalize NRP1 levels and decrease the ability of metastatic and drug-resistant cells to respond to growth factor stimulation (Fig. 7). These findings add a mechanistic framework to support the therapeutic concept of combining BET inhibitors or other epigenetic modulators with FGFR-targeted compounds [52–55].

Overall, our studies present a broad characterization of gene expression changes upon treatment and 3D acquisition of resistance to ErbB-targeted therapies. Moreover, we present a novel dataset that identifies changes in FGFR1 protein:protein interactions as cells undergo EMT. Use of these discovery approaches led us to investigate the molecular interaction between NRP1 and FGFR1. Our findings suggest that antibody-mediated targeting of cell surface NRP1 has therapeutic limitations, but whole-cell expression of the protein can be normalized using BET inhibitors. Combined use of epigenetic modulators in combination with targeted inhibition of FGFR presents a potential approach to control the growth of ErbBi-resistant breast cancer metastasis.

## Materials and methods

### Cell lines and reagents

The D2.A1, HMLE, NMUMG, and MCF-10A cell lines were constructed to stably express firefly luciferase via stable transfection under zeocin or blasticidin selection. D2.A1 cells were cultured in Dulbecco’s modified Eagle’s medium (DMEM) supplemented with 10% fetal bovine serum and 1% Pen/Strep as described previously [56]. MCF-10A cells were maintained as described previously [29]. The HMLE cells were transformed by overexpression of HER2 via stable transduction under puromycin selection, yielding the parental HME2 cell line. In order to establish resistance to ErbB-targeted therapies, HME2 cells were treated with lapatinib to generate LAPR cell populations [29]. HME2 cells were engrafted onto the mammary fat pad of immunocompromised mice and subsequent BMs were subcultured, yielding the HME2-BM cell line [12]. HME2-BM cells were treated with either afatinib or neratinib for 4 weeks to generate afatinib-resistant (BMAR) and neratinib-resistant (BMNR) cell lines. The NMuMG cells were transformed by overexpression of EGFR via stable viral transduction under puromycin selection as described previously [57]. Once established, all drug-resistant cell lines were maintained in the absence of drug in DMEM supplemented with 10% fetal bovine serum, 1% Pen/Strep and 10.5 μg/ml insulin. Manipulation of NRP1 expression in the D2.A1 cells was achieved through lentiviral-mediated transduction of TRCN0000029859, TRCN0000029863, or a scrambled control shRNA in the pLKO.1 vector (GE Dharmacon, Lafayette, CO). Overexpression of Twist in HMLE cells; MCF-10A and HMLE and NMuMG cells was accomplished via stable transduction under puromycin selection. All cell lines were authenticated and tested for mycoplasma contaminated via R&D Systems MycoProbe Mycoplasma Detection Kit. The anti-NRP1 antibody YW107.4 was kindly provided by Genentech [34]. FGF2, PDGFa, and HGF were purchased from GoldBio (St. Louis, MO). JQ1 was obtained from Caymen Chemical (Michigan, USA).

### Animal models

All in vivo assays were conducted under IACUC approval from the Purdue University. Luciferase-expressing D2.A1 (1 × 10^6^/100 µl) cells were injected into the lateral tail vein of female 8-week-old Balb/C mice. For inhibitor treatments, groups were randomized based on initial bioluminescent readings. Sample sizes were chosen based on previous experiments using this approach and no blinding was done. Pulmonary tumor growth was subsequently quantified by bioluminescence at the indicated time points using the Advanced Molecular Imager (AMI) (Spectral Instruments, Tucson, AZ). Lungs from all animals were removed upon necropsy and fixed in 10% formalin and dehydrated in 70% ethanol, sectioned, and stained with hematoxylin and eosin for visualization of pulmonary metastatic nodules.

### Immunological assays

For immunoblot analyses, cells were lysed using a modified RIPA lysis buffer containing 50 mM Tris, 150 mM NaCl, 0.25% sodium deoxycholate, 1.0% NP40, 0.1% sodium dodecyl sulfate (SDS), protease inhibitor cocktail, 10 mM activated sodium orthovanadate, 40 mM β-glycerolphosphate, and 20 mM sodium fluoride. These lysates were separated by reducing SDS-polyacrylamide gel electrophoresis and probed for NRP1 (R&D System; AF566), FGFR1 (Cell Signaling; 9740), phospho-ERK1/2 (Cell Signaling; 9101), total ERK1/2 (Cell Signaling; 4695), FN (BD Biosciences; 610078), vimentin (BD Biosciences, 550513), twist (Abcam, ab50887), E-Cad (BD Biosciences, 610182), or β-tubulin (DSHB, E7-s).

For co-IP, NMuMG cells overexpressing FGFR1-GFP were stably transfected with NRP1 constructs. FGFR1 was pulled down using the GFP-trap protocol (ChromoTek). Associated proteins were characterized by mass spectroscopy or directly blotted for NRP1. The endogenous FGFR1 and NRP1 interaction was confirmed in D2.A1 cells using an FGFR1 antibody or rabbit immunoglobulin G in conjunction with protein A/G agarose beads.

For colocalization studies, NMuMG cells stably expressing FGFR1-GFP were transiently transfected with an NRP1-mcherry construct in the presence or absence of TGF-β1 (5 ng/ml) for 4 days. For visualizing FGF2 activation of phospho-ERK1/2, D2.A1 cells were grown and processed on 3D culture scaffolds as described previously [35]. Briefly, cells were fixed in 4% paraformaldehyde, permeabilized in 0.1% Triton X-100 and processed with phospho-ERK1/2 (Cell Signaling, 9101). Cells were mounted and imaged using a Zeiss upright confocal microscope.

### Cell viability and proliferation assays

The viability of cells was measured using the CellTiter-Glo assay (Promega) according to the manufacturer’s protocol. For the cells stably expressing firefly luciferase, the activity of luciferase was also monitored using a cell-permeable luciferin (GoldBio) as an indicator of cell number.

### 3D spheroid assay

BMAR, LAPR, and D2.A1 cells (2–5 × 10^3^) were plated in a non-adherent round-bottom 96-well plate in full growth media. The cells were cultured for 7 days, after which tumorspheres were physically transferred along with growth media to a flat-bottom 96-well plate pre-coated with 50 μl of cultrex (Trevigen, Gaithersburg, MD). After 24 h, culture media containing the indicated growth factors in the presence or absence of JQ1 was replaced. Tumor spheroids were grown for an additional 8 days for D2.A1 cells or 14 days for BMAR and LAPR cells at the indicated time points; structure size was visualized by brightfield imaging and quantified using an R language program developed by our lab.

### mRNA analyses

Total RNA was isolated (Omega Bio-Tek, Norcross, GA) and global gene expression changes in the NME cells upon acquisition of resistance to AG1478 were determined using the Affymetrix Mouse gene 1.0 ST microarray platform as described in GSE140978. Gene expression changes in the HME2 cells upon acquisition of resistance to lapatinib were determined using the NanoString PanCancer progression gene panel (Supplementary Table 1). For reverse transcription-polymerase chain reaction (RT-PCR), RNA was reverse-transcribed (Thermo Fisher), and semi-quantitative real-time PCR was performed using iQ SYBR Green (Thermo Fisher). Specific detection of NRP1 was done using the following primers: mouse, 5′-GGCACAGGTGATGACTT-3′ and 5′-GTCAGCACACTCCACCT-3′; human, 5′-ATGACGACCAGGCCAACTG-3′ and 5′-TTGATACCTGATTGTAGGTGCTG-3′. These data were normalized to glyceraldehyde 3-phosphate dehydrogenase.

### Identification of transcriptional mediators of NRP1 expression using METABRIC

Median normalized data were downloaded for the Breast Invasive Carcinoma primary solid tumor cohort dataset [Broad, 2016] from the firebrowse website (http://firebrowse.org/, Broad), filtered for NRP1- and EMT-related genes [58]. Finally, the correlation between genes was calculated using the Spearman metric in the R language and environment for statistical computing [R Core Team, 2019].

### Statistical analyses

Either two-way analysis of variance or a two-sided *t* tests were used where the data met the assumptions of these tests and the variance was similar between the two groups being compared. *P* values <0.05 were considered significant. No exclusion criteria were used for these studies.

## Supplementary information

Supplemental Table 1

Supplemental Table 2

Supplemental Table 3

Supplemental Table 4

Supplemental Figure legends

Figure S1

Figure S2

Figure S3

Figure S4

Figure S5

Figure S6

Figure S7

Figure S8
