# Correction: Epigenetic targeting of neuropilin-1 prevents bypass signaling in drug-resistant breast cancer

**DOI:** 10.1038/s41388-023-02654-1

**Published:** 2023-03-15

**Authors:** Ammara Abdullah, Saeed Salehin Akhand, Juan Sebastian Paez Paez, Wells Brown, Li Pan, Sarah Libring, Michael Badamy, Emily Dykuizen, Luis Solorio, W. Andy Tao, Michael K. Wendt

**Affiliations:** 1 Department of Medicinal Chemistry and Molecular Pharmacology, Purdue University, West Lafayette, IN 47907 USA; 2 Department of Biomedical Engineering, Purdue University, West Lafayette, IN 47907 USA; 3 Purdue University Center for Cancer Research, Purdue University, West Lafayette, IN 47907 USA

**Keywords:** Breast cancer, Cancer imaging, Targeted therapies, Oncogenes, Target identification

Correction to: *Oncogene* (2020) 40:322–333 10.1038/s41388-020-01530-6, Published online: 30 October 2020

The original online version of this article was revised:

The Tubulin loading controls in Supplementary Figure 7 panel A were mistakenly duplicated in panel D.

The Supplementary Figure 7 was corrected. The authors apologize for this oversight.

The original article has been corrected.

