# Supplementary material for: Epigenetic targeting of neuropilin-1 prevents bypass signaling in drug-resistant breast cancer
Source: Oncogene. 2020 Oct 30;40(2):322–33. doi: 10.1038/s41388-020-01530-6 (PMC7808937; doi:10.1038/s41388-020-01530-6)
Supplement: Supplementary file 5 — Supplemental Figure legends [file 41388_2020_1530_MOESM5_ESM.docx]

**Epigenetic targeting of neuropilin-1 prevents bypass signaling in
drug resistant breast cancer**

Ammara Abdullah^1^, Saeed Salehin Akhand^1^, Juan Sebastian Paez Paez^1^, Wells Brown^1^, Li Pan^1^, Sarah Libring^2^, Michael Badamy^1^, Emily Dykuizen^1,3^, Luis Solorio^2,3^, W. Andy Tao^1,3^, and
Michael K. Wendt^1,3*^

**Supplemental Figure Legends:**

**Supplemental Figure 1.** The drug resistant phenotype of the AGR cell line is stable following *in vitro* expansion and is independent of EGFR signaling. **(a)** NME and AGR cells were grown in 3D environment under non-stimulated (NS) and AG1478 (1μM) conditions for 10 days. The viability of cells was calculated Cell-titer Glo assays. Data normalized to control-NME cells and are the mean ±SD of three independent experiments resulting in the indicated p-value. **(b)** Representative 3D growth images of the NME and AGR cell lines as described in panel a. **(c)** NMuMG, NME and AGR cells were stimulated with 50 ng/ml of EGF (E) for 30 minutes in the presence or absence of 1μM of the EGFR inhibitor AG1478 (AG). Phosphorylation of EGFR and its downstream targets, ERK1/2 and STAT3 were determined while total expression levels of these proteins served as loading controls. Data in panels b and c are representative of at least three independent experiments.

**Supplemental Figure 2.** NRP1 expression is enhanced upon acquired resistance to ErbBi therapy. **(a-b)** Immunoblot analyses showing NRP1 expression in HME2 drug sensitive (parental and bone metastases (BM)) and the corresponding lines selected for resistance via prolonged treatment with lapatinib (LAPR), neratinib (BMNR), or afatinib (BMAR). **(c)** Expression of NRP1 mRNA in the drug sensitive vs. resistant cell lines using RT-PCR. The relative mRNA expression was normalized to the parental cell line. Data are the mean ±SE of triplicate experiments. **(d)** BreastMark combined survival analysis of 74 patients bearing HER2-enriched primary tumors as determined by the PAM50 that had been previously treated with chemotherapy. Patients were divided into two groups based on the median expression value of NRP1 and differential survival was analyzed by a log rank test, resulting in the indicated *P*-value and hazard ratio.

**Supplemental Figure 3.** The D2.A1 model of inherent resistance to ErbB inhibition. **(a)** The nonmetastatic D2.OR cells and their isogenic, metastatic counterpart D2.A1 cells were treated with the indicated concentrations of the ErbB inhibitor neratinib for 72 hours at which point cell viability was determined. **(b)** The IC50 values for each cell line was determined over three independent dose response assays as described in panel A, resulting in the indicated mean, ±SD, and p-value. **(c)** The D2.OR and D2.A1 cells were stimulated with EGF for 30 minutes and analyzed for phosphorylation of EGFR, HER2, and ERK1/2. Total levels for these proteins and β-tubulin served as a loading control. Data are representative of three independent assays.

**Supplemental Figure 4.** Antibody-mediated targeting of NRP1 increases pulmonary tumor growth. **(a)** Bioluminescent quantification of D2.A1 control (scram) and NRP1 depleted (shNRP1-01 and -02) cell growth in 2D culture. Data are the mean ± SE of triplicate experiments where * indicates p<0.05 using Dunnett’s modified t-test. **(b)** Bioluminescent quantification of D2.A1 cell growth in 3D culture treated with control IgG antibody or anti-NRP1 antibody YW107.7 (2mg/mL) in the presence or absence (NS) of FGF2 (20ng/ml). Data are the mean ±SE of triplicate experiments resulting the indicated *P*-value. **(c)** Bioluminescent quantification of D2.A1 pulmonary tumor growth in IgG treated animals (No Drug) and those treated with the YW107.7 antibody 10mg/kg/Q.O.D (*P*-value = 0.0257) **(d)** Representative bioluminescent images for the control (No Drug) and YW107.7 antibody-treated groups at the indicated times.

**Supplemental Figure 5.** NRP1 is required for HGF and PDGF-mediated cell growth. **(a)** Time-course experiment of HGF (50ng/mL) and PDGF (100ng/mL) induced phosphorylation of ERK1/2 and AKT in the D2.A1 cells. **(b)** Immunoblot analyses showing differential phosphorylation of ERK1/2 and AKT stimulated by HGF (50ng/ml) and PDGF (100ng/ml) in control (scram) and NRP1 depleted (sh-02) D2.A1 cells. **(c-d)** Bioluminescent quantification of control (scram) and NRP1 depleted (sh-02) D2.A1 cells growing under nonstimulated (NS) 3D culture conditions or in the presence of HGF (50ng/mL) or PDGF (100ng/mL). Data are the mean ±SE of triplicate experiments resulting the indicated *P*-values.

**Supplemental Figure 6.** Twist-driven expression of NRP1 can be blocked via inhibition of BET proteins. **(a)** Chromatin immuno-precipitation for BRD4 or H3K27 acetylation (ac) followed by DNA sequencing in SUM147 cells. Data indicate that BRD4 is bound to H3K27 in the region immediately upstream of NRP1 transcriptional start site. **(b)** Analysis of GSE63584 RNA sequencing data indicate that a 12 hour treatment of the SUM147 cells with JQ1 (500 nM) decreases expression of NRP1. **(c)** Analysis of GSE53222 RNA sequencing data suggest that overexpression of Twist is sufficient to increase NRP1 expression in HMLE cells, and that this can be inhibited by a 6 hour treatment with JQ1. Data are from a single replicate.

**Supplemental Figure 7.** Acute and chronic JQ1 treatment inhibits NRP1 expression and inhibits growth factor signaling. **(a)** Immunoblot analyses showing expression of NRP1 and FGFR1 proteins in LAPR cells following 7 days of treatment with JQ1 (10nM). **(b)** RT-PCR showing expression of NRP1 transcripts in LAPR cells following 7 days of treatment with JQ1 (10nM) treatment. **(c)** RT-PCR showing expression of NRP1 transcripts in BMAR cells following 6 hours of treatment with JQ1 (500nM). **(d)** Immunoblot analyses showing expression of NRP1 and FGFR1 protein in D2.A1 cells following 7 days of treatment with JQ1 (10nM). (e) Immunoblot analyses showing differential phosphorylation of ERK1/2 (p-ERK1/2) upon FGF2 (20ng/ml) and PDGF (100ng/mL) stimulation in D2.A1 cells following 6 hours of treatment with JQ1 (500nM). Expression of total ERK1/2 (tERK1/2) served as loading control.

**Supplemental Figure 8.** JQ1 inhibits growth factor-induced tumor growth and invasion. **(a)** BMAR and D2.A1cell lines were treated with the indicated concentrations of JQ1 for 96 hours and cell viability was determined. Data are the mean ±SE of triplicate experiments. **(b)** Bioluminescent quantification of control and JQ1-treated D2.A1 cells growing under 2D culture conditions in the presence or absence (NS) of FGF2 (20ng/ml). FGF2 was added at 3 days after initiation of JQ1 treatment, and experiment was terminated at day 7. Data are the mean ±SE of triplicate experiments resulting the indicated *P*-values. **(c)** Phase contrast images showing 3D cell invasion of D2.A1 cells under non-stimulated (NS) conditions or in response to HGF (50ng/ml), PDGF (100ng/ml), and FGF2 (20ng/ml) in the absence (DMS) or presence of JQ1 (25nM). **(g)** Quantification of D2.A1cell invasion into the surrounding matrix, 8 days after spheroids were transferred to cultrex where * indicated p<0.05 vs. JQ-1 using Sidak’s modified t-test. The number of invaded/migrated cells were counted for two images per treatment.
