# Supplementary figures and images for: Epigenetic targeting of neuropilin-1 prevents bypass signaling in drug-resistant breast cancer
Source: Oncogene. 2020 Oct 30;40(2):322–33. doi: 10.1038/s41388-020-01530-6 (PMC7808937; doi:10.1038/s41388-020-01530-6)

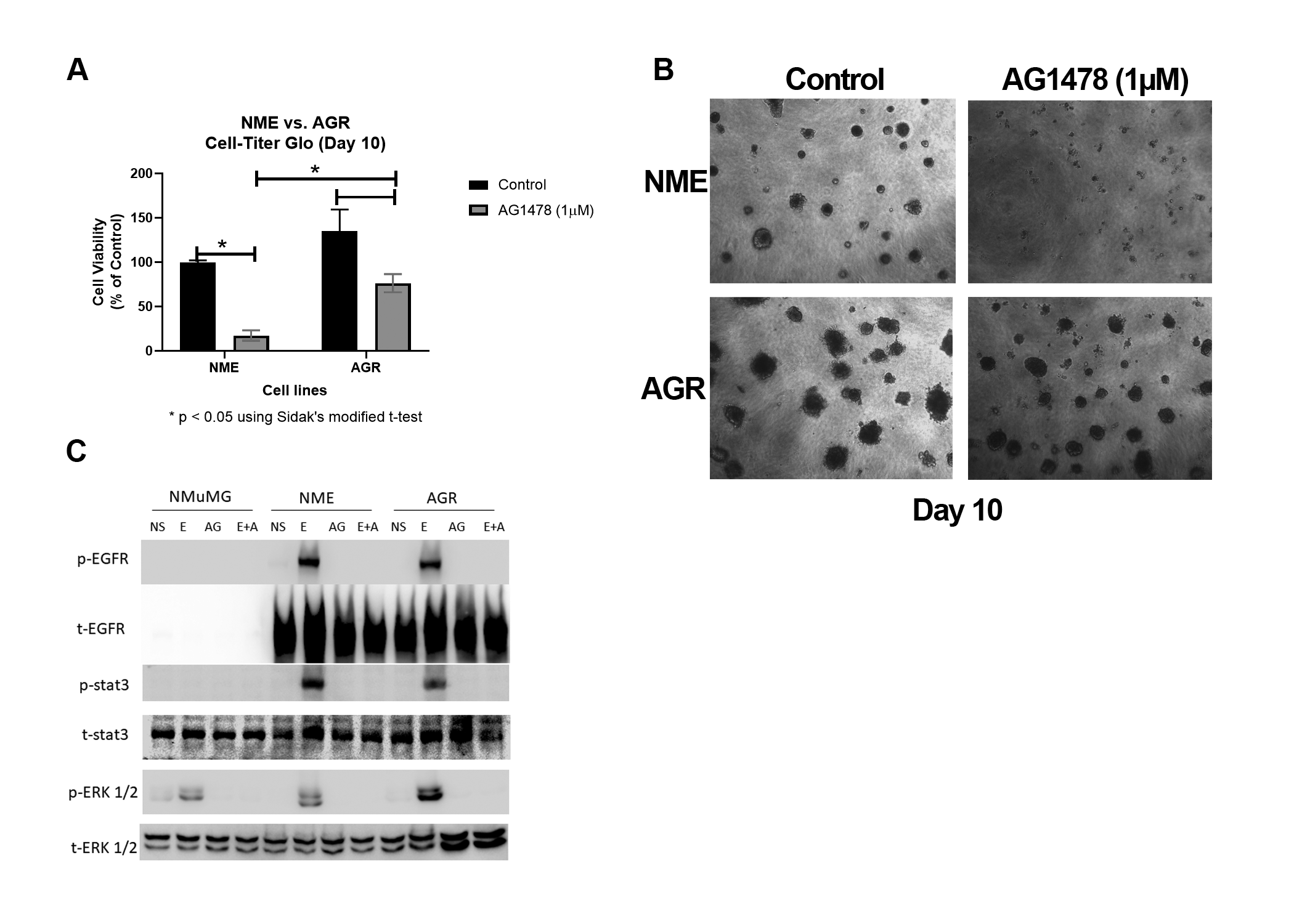

Supplement: Supplementary file 6 — Figure S1 [file 41388_2020_1530_MOESM6_ESM.tif]

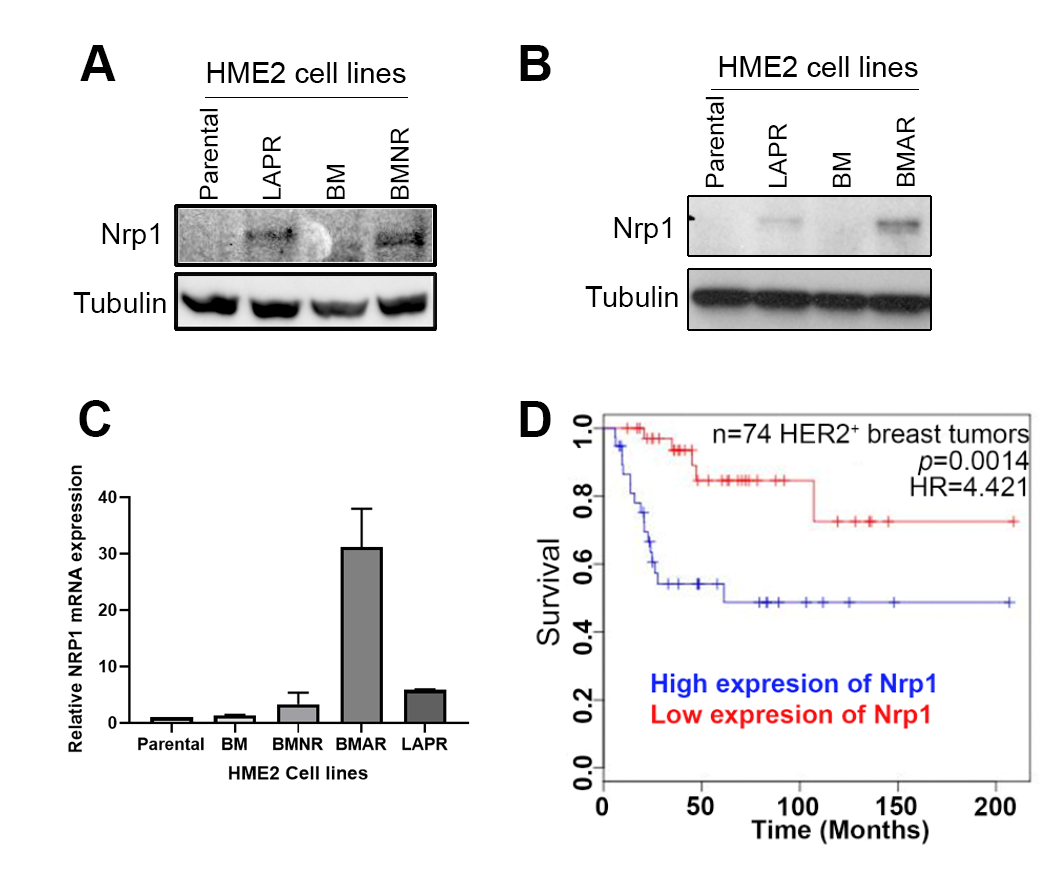

Supplement: Supplementary file 7 — Figure S2 [file 41388_2020_1530_MOESM7_ESM.tif]

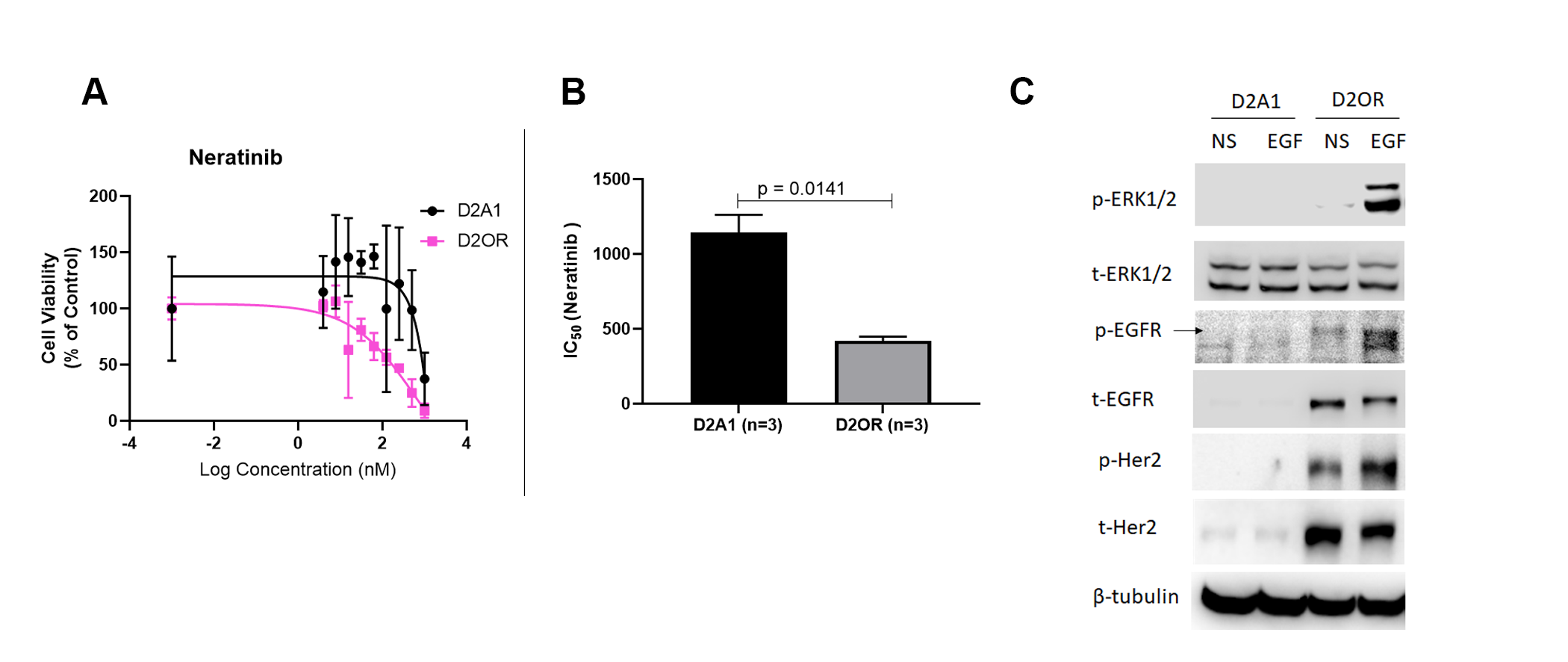

Supplement: Supplementary file 8 — Figure S3 [file 41388_2020_1530_MOESM8_ESM.tif]

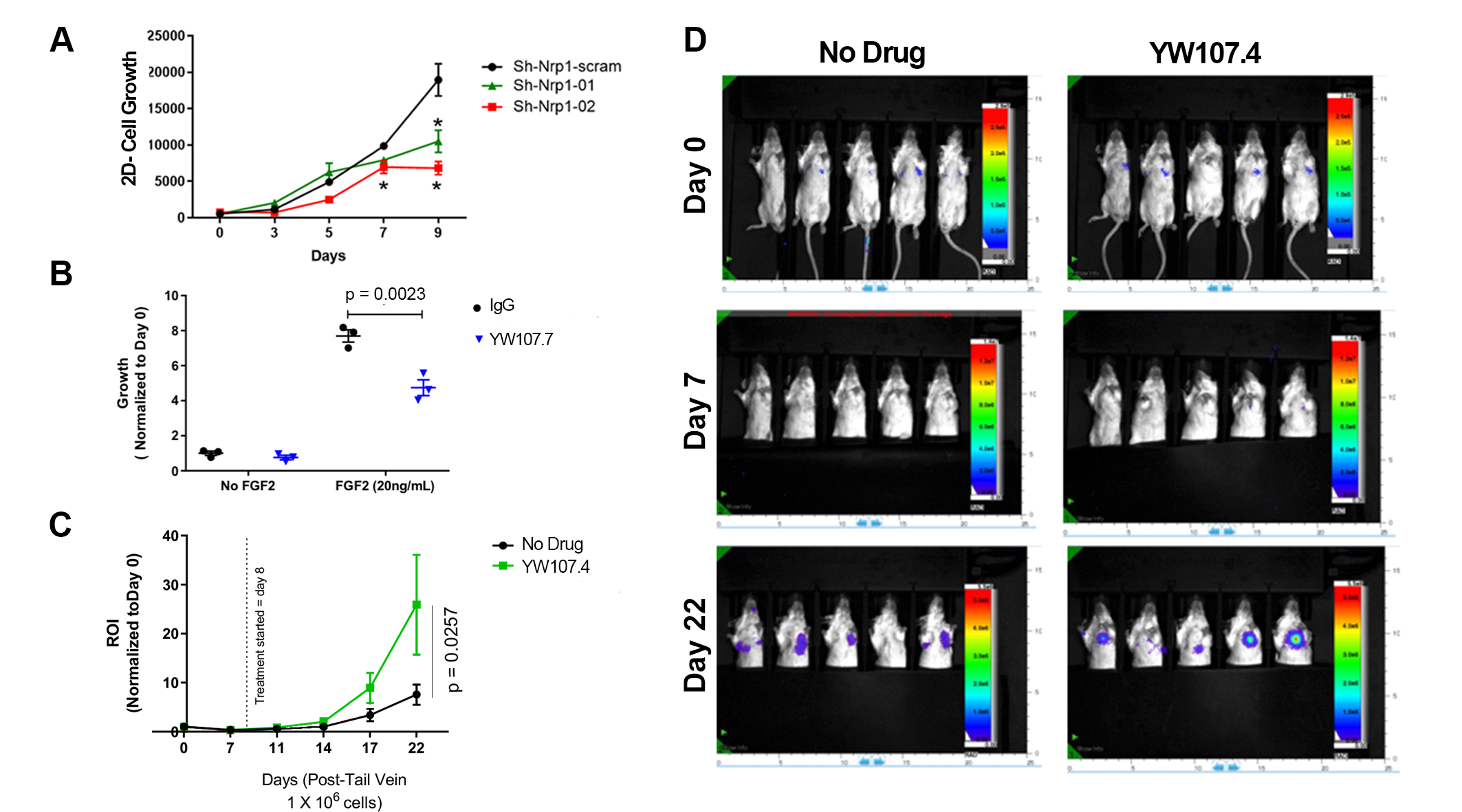

Supplement: Supplementary file 9 — Figure S4 [file 41388_2020_1530_MOESM9_ESM.tif]

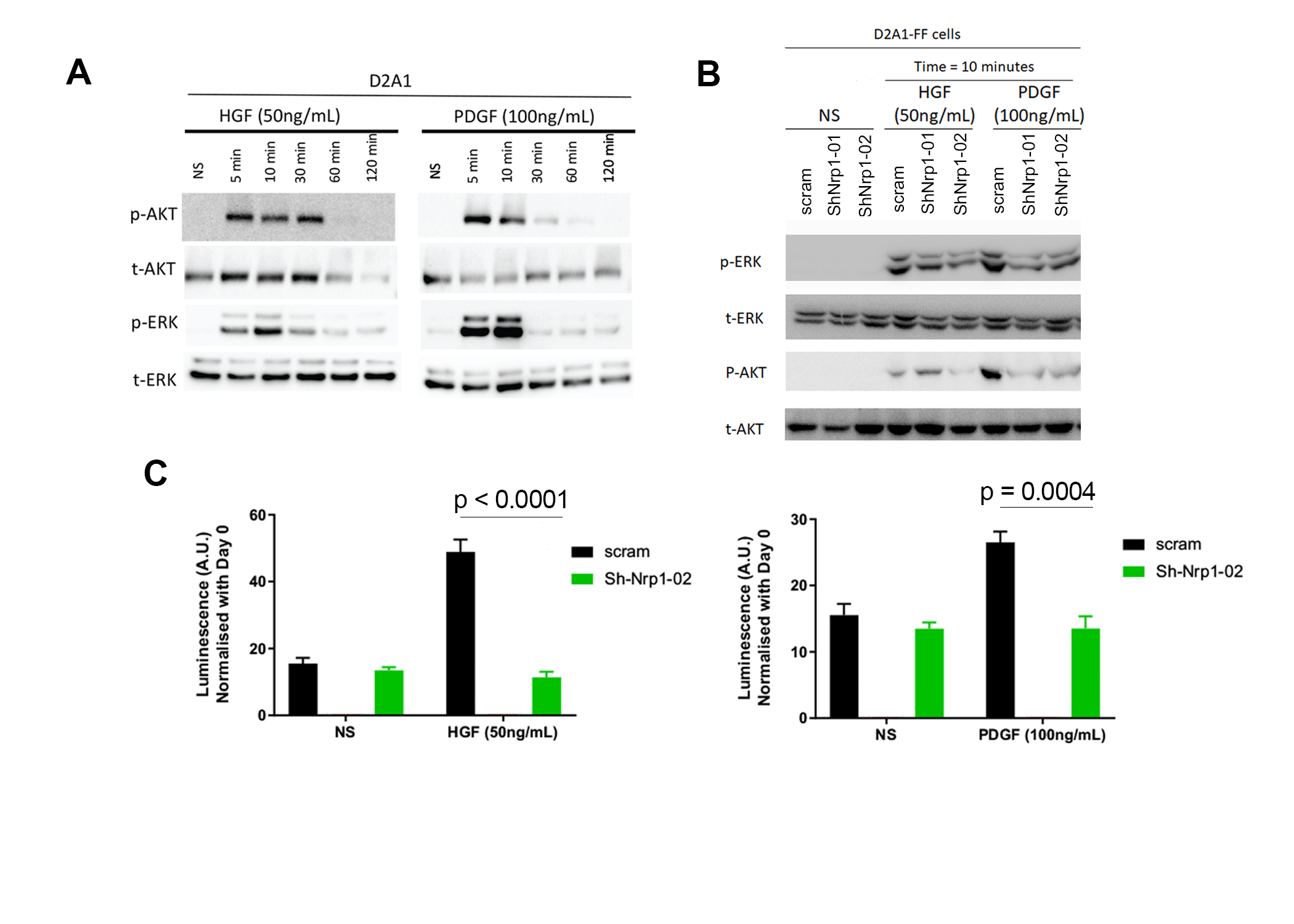

Supplement: Supplementary file 10 — Figure S5 [file 41388_2020_1530_MOESM10_ESM.tif]

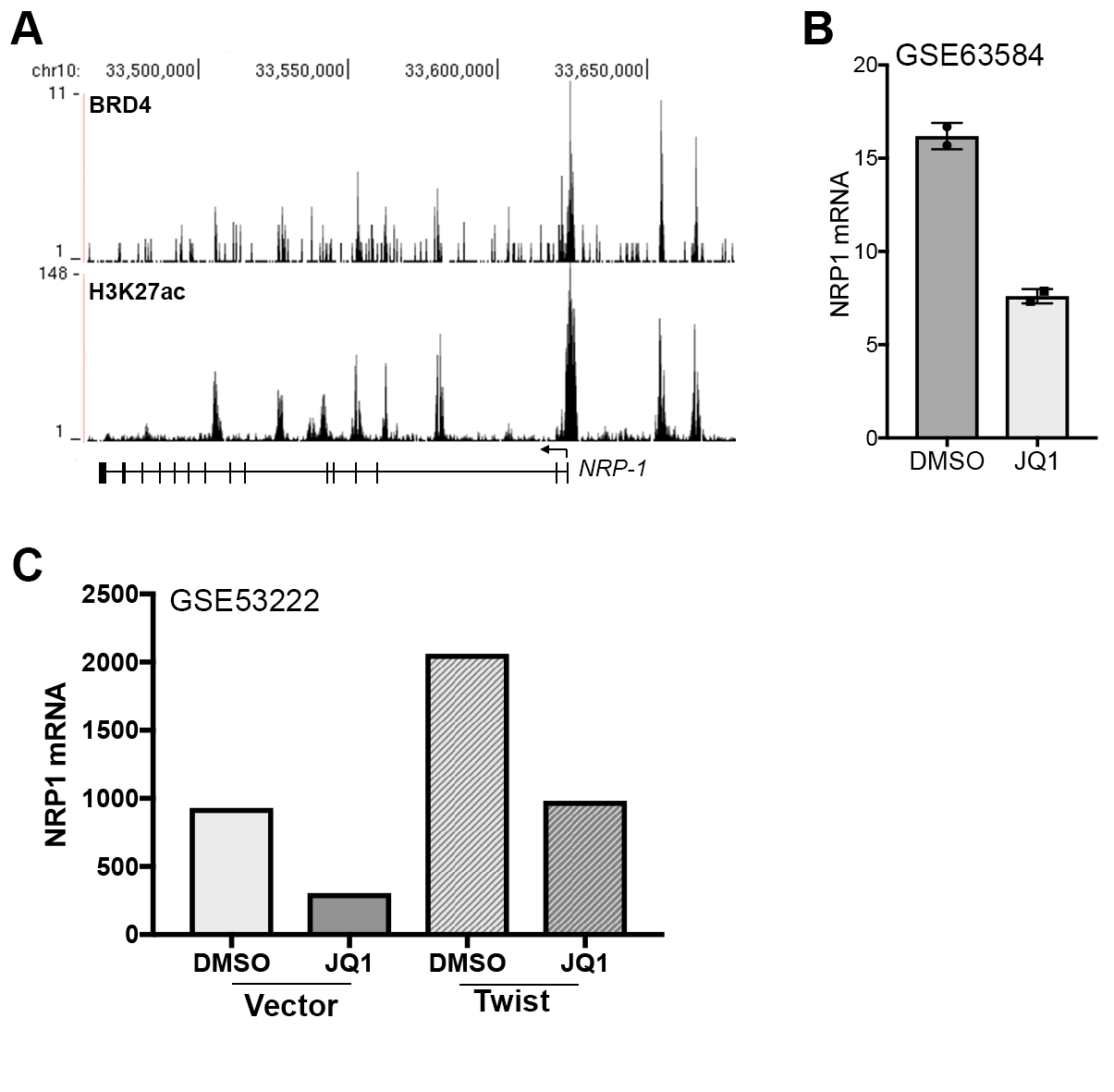

Supplement: Supplementary file 11 — Figure S6 [file 41388_2020_1530_MOESM11_ESM.tif]

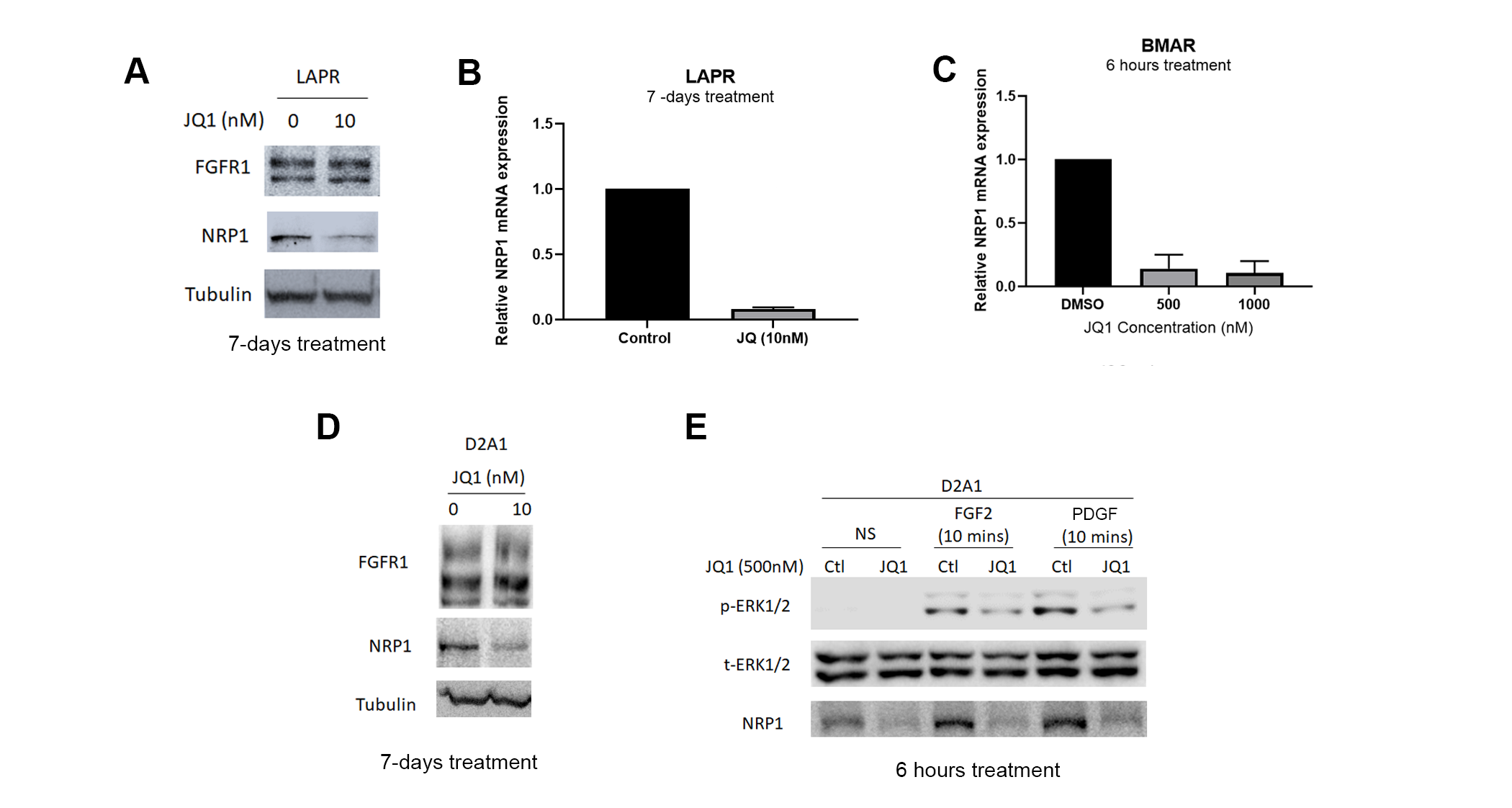

Supplement: Supplementary file 12 — Figure S7 [file 41388_2020_1530_MOESM12_ESM.tif]

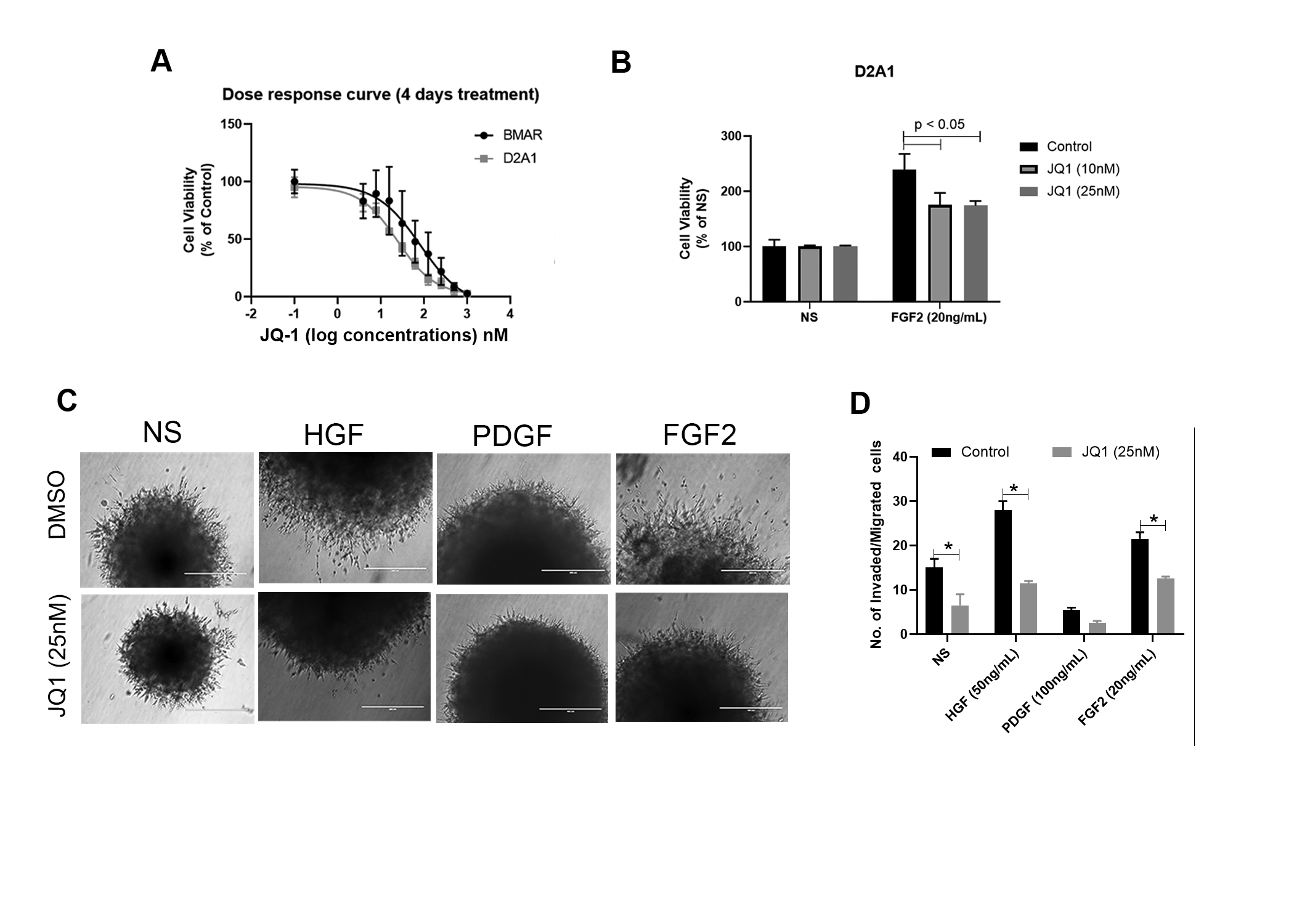

Supplement: Supplementary file 13 — Figure S8 [file 41388_2020_1530_MOESM13_ESM.tif]
